# Modulation of *ERG* gene expression in fluconazole-resistant human and animal isolates of *Trichophyton verrucosum*

**DOI:** 10.1007/s42770-021-00585-1

**Published:** 2021-08-05

**Authors:** Sebastian Gnat, Dominik Łagowski, Mariusz Dyląg, Aneta Ptaszyńska, Aneta Nowakiewicz

**Affiliations:** 1 Department of Veterinary Microbiology, Faculty of Veterinary Medicine, Institute of Preclinical Veterinary Sciences, University of Life Sciences, Akademicka 12, 20-033 Lublin, Poland; 2 Department of Mycology and Genetics, Faculty of Biological Sciences, Institute of Genetics and Microbiology, University of Wroclaw, Wroclaw, Poland; 3 Department of Imm, unobiology, Institute of Biological Sciences, Faculty of Biology and Biotechnology, Maria Curie-Skłodowska University, Lublin, Poland

**Keywords:** *ERG* genes, Expression profile, Fluconazole, Resistance, *Trichophyton verrucosum*

## Abstract

Dermatophytes are a group of eukaryotic microorganisms characterized by high capacity to colonize keratinized structures such as the skin, hair, and nails. Over the past years, the incidence of infections caused by zoophilic species, e.g., *Trichophyton verrucosum*, has been increasing in some parts of the world, especially in Europe. Moreover, the emergence of recalcitrant dermatophytoses and in vitro resistant dermatophytes has become a cause of concern worldwide. Here, we analyzed the mechanisms underlying resistance to fluconazole among clinical isolates of *T. verrucosum.* Quantitative RT-PCR was carried out to determine the relative expression levels of mRNA transcripts of *ERG3*, *ERG6*, and *ERG11* genes in the fungal samples using the housekeeping gene GAPDH as a reference. Our results showed that the upregulation of the *ERG* gene expression is a possible mechanism of resistance to fluconazole in this species. Furthermore, *ERG11* is the most statistically significantly overexpressed gene in the pool of fluconazole-resistant *T. verrucosum* isolates. Additionally, we have demonstrated that exposure to fluconazole increases the levels of expression of *ERG* genes in fluconazole-resistant isolates of *T. verrucosum*. In conclusion, this study has shown one of the possible mechanisms of resistance to fluconazole among zoophilic dermatophytes, which involves the maintenance of high levels of expression of *ERG* genes after drug exposure.

## Introduction

Dermatophytes are a group of primarily pathogenic filamentous fungi classified in the Arthrodermataceae family, within the order Onygenales [1]. These eukaryotic microorganisms are characterized by a high capacity to attack keratinized structures such as the skin, hair, and nails in humans and animals causing mainly superficial cutaneous infections [2]. Indeed, a biological characteristic of dermatophytes is their ability to invade keratin-rich tissues by producing enzymes [3]. *Trichophyton rubrum* is the commonest causative agent with a diagnosis frequency comparable to the state of a global epidemic [4]. However, the incidence of infections caused by zoophilic dermatophytes *T. mentagrophytes* [2, 5, 6] and less common *T. verrucosum* is increasing in some parts of the world [7–11]. Although most of the literature data indicate the occurrence of infections caused by these species of dermatophytes in animals, zoonoses of these etiologies have been reported in humans professionally involved in animal husbandry [9, 12, 13]. Noteworthy, the reverse zoonosis caused by *T. verrucosum* has also been reported in Europe [8].

The emergence of recalcitrant dermatophytoses and in vitro resistant dermatophytes over the past few years has become a cause of concern worldwide [2, 14, 15]. Consequently, this has led to renewed research interest in dermatophytoses, one of the commonest fungal infections of humans and animals [16, 17]. However, molecular mechanisms underlying the reduced susceptibility to antifungals are not fully described or clearly elucidated.

Antifungal drugs commonly used in the treatment of dermatophytoses largely interfere with the different steps of the ergosterol synthesis pathway [14]. Ergosterol is an essential component of fungal plasma membranes [18]. Azoles, including fluconazole which is the main object of the present study, inhibit lanosterol 14α-demethylase (CYP51A1), i.e., a cytochrome P450-dependent enzyme, causing ergosterol depletion and disturbing the fluidity, permeability, and activity of membrane-associated proteins [19]. Azole-resistant phenotypes of dermatophytes are usually related to point mutations in the *ERG11* (encoding the enzyme lanosterol 14-α demethylase) or *ERG3* (encoding the enzyme sterol C-5 desaturase) and *ERG6* (encoding the enzyme sterol C-24 methyltransferase) genes, their duplication conferring overexpression of target enzymes, or mutations in the cytochrome P450-encoding gene affecting drug binding [20]. Another mechanism of azole resistance is connected with overexpression of genes related with ATP binding cassette (ABC) transporters responsible for drug efflux [21].

In the last few years, the number of azole-resistant clinical isolates from the *Trichophyton* genus and the pool of infected patients who have failed to respond to the initial therapy for such infections have increased [2, 15, 22]. However, patient insensitivity to azole treatment has only been reported in cases caused by the anthropophilic dermatophyte *T. rubrum* [21]*.* In turn, azole-resistant phenotypes have never been found among zoophilic dermatophytes, particularly *T. verrucosum*.

In the present study, we evaluated the mechanisms underlying the sensitivity and resistance to fluconazole among clinical isolates of *T. verrucosum* obtained from human and animal dermatophytoses in Poland. The quantitative real-time PCR (qRT-PCR) technique was employed to examine the transcriptional modulation of *ERG* genes.

## Materials and methods

### Clinical dermatophyte isolates

In total, 25 clinical isolates of fluconazole-resistant *Trichophyton verrucosum* were obtained from humans (*n* = 10) and animals (*n* = 15) with typical symptoms of dermatomycosis from different regions of Poland (Table 1). The dermatophyte strains were isolated from outbreaks of zoophilic mycoses noted from 2011 to 2019. All human cases were documented as zoonoses. All of these isolates showed susceptibility to azoles, excluding fluconazole, and toward allylamine-type drugs. Additionally, five clinical isolates of *T. verrucosum* with sensitivity to fluconazole (MIC < 1 μg/ml) obtained from cattle with ringworm were used as controls.

**Table. 1:** Isolates of dermatophytes used in the study with description, results of fluconazole sensitivity, and *ERG* gene expression analysis

Host		Isolates	Type of infection/localization of skin lesions	Accession numbers of ITS sequences	MIC of fluconazole [µg/ml]	MIC interpretation*	*ERG* gene expression analysis**					
							Exposed to fluconazole			Without exposure to fluconazole		
							*ERG3*	*ERG6*	*ERG11*	*ERG3*	*ERG6*	*ERG11*
Human		TV16	Kerion celsi/head	MG251681	64	R	2.9	2.9	2.8	2.1	2.4	2
		TV23	Kerion celsi/head	MG251688	64	R	1.9	1.8	2.9	1.15	1.3	2.1
		TV28	Kerion celsi/head	MG251693	128	R	2.2	2	2.9	1.9	1.5	2.1
		LL7	Tinea unguium	MK369715	8	R	2.3	2.1	3	1.7	1.4	1.9
		TV35	Tinea capitis	MT808415	32	R	2.4	2	3.1	1.9	1.6	2.2
		TV36	Tinea capitis	MT808416	32	R	2.3	2.2	3.1	1.7	1.5	2.4
		TV46	Tinea capitis	MT808417	8	R	2.2	2.4	3.9	1.6	1.3	2.6
		TV49	Tinea capitis	MT808418	64	R	2.6	2	3.8	1.7	1.5	2.7
		TV51	Tinea capitis	MT808419	16	R	2.4	2.9	3.8	1.6	1.3	2.9
		TV56	Tinea capitis	MT808420	32	R	2.3	2.1	3.1	1.4	1.2	2.1
Animals	Cattle	TV22	Head, thorax	MG251687	128	R	2.5	2.7	3.2	1.3	1.5	2
	Cattle	TV24	Head, thorax	MG251689	128	R	2.5	1.9	3.5	1.3	1.3	2.8
	Sheep	TV25	Head	MG251690	64	R	2.6	1.9	3.3	1.5	1.6	2.5
	Sheep	TV26	Head	MG251691	64	R	2.7	2.1	3.7	1.4	1.7	2.2
	Cattle	TV27	Head, thorax	MG251692	128	R	2.7	2.2	3.5	1.3	1.4	2.3
	Cattle	TV8	Head, thorax	MG251673	32	R	2.8	2.2	3.8	1.8	1.4	2.5
	Cattle	TV1	Head, thorax	MG251666	8	R	2.2	2	2.9	1.5	1.3	2
	Sheep	TV20	Head	MG251685	64	R	2.5	1.9	2.1	1.4	1.3	2.1
	Llama	LL1	Head, neck	MK369717	4	R	2.1	2.1	2.5	1.2	1.2	1.9
	Llama	LL2	Head, neck	MK369716	64	R	2.7	2.1	3	1.4	1.4	2.1
	Alpaca	ALV12	Neck	MN960481	2	R	2.4	2	2.9	1.5	1.5	2.3
	Alpaca	ALV32	Trunk	MN960484	64	R	2.7	1.9	3.1	1.6	1.5	2
	Calves	TV38	Multiple site	MT808618	32	R	2.7	2.6	3.4	1.3	1.8	1.9
	Calves	TV45	Multiple site	MT808619	64	R	2.8	1.9	3	1.4	1.5	1.9
	Calves	TV50	Head	MT808620	32	R	2.4	1.9	3.2	1.2	1.2	2.1
	Cattle	TVS91	Neck	MT818238	0.5	S	2	2	2.4	1.1	1.5	1.1
	Cattle	TVS92	Thorax	MT818239	0.5	S	1.7	1.5	2.3	1.2	1.2	1.9
	Cattle	TVS93	Head, neck	MT818240	0.25	S	1.9	1.45	2.9	1.4	0.9	0.9
	Cattle	TVS94	Thorax	MT818241	0.5	S	2.2	1.6	2.1	1.5	1.2	1.1
	Cattle	TVS95	Head	MT818242	0.5	S	2.9	1.6	2.2	2	1.3	1.2

^*^R resistant, *S* susceptible; **geometric mean of the mRNA fold change for *ERG* genes normalized relative to *GAPDH:* standard deviation between replicates for each isolate did not exceed 5%

**Fig. 1:**
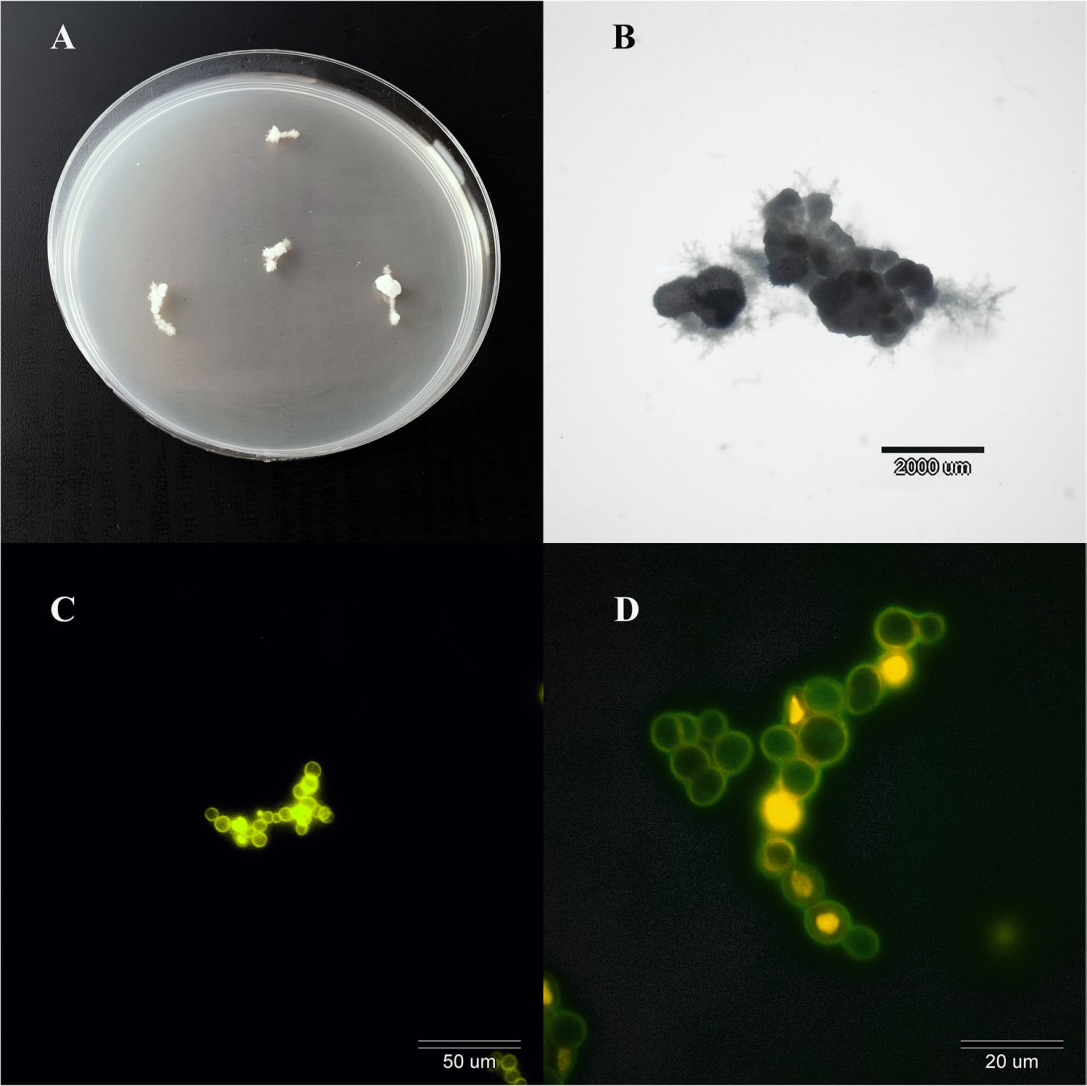
Micro- and macroscopic morphology of *Trichophyton verrucosum* after 20 days of incubation. A—Beige colonies with a friable texture and a yellow or yellow-orange reverse. The size of the colony in the range from 5 to 8 mm. The edges of the colony strongly corrugated and the image of the obverse and the reverse reminiscent of a cauliflower or a rotor of a turbine. B—Colony in a magnification × 10 (Olympus SZ61, Tokyo, Japan). C—The micromorphological image on the microscope slides exhibit circular chlamydospores. The macroconidia have not been observed (Olympus BX51, Tokyo, Japan, magnification × 400). D—The chlamydospores in a magnification × 1000

Species identification analysis was performed as described previously by Gnat et al. [23]. All clinical isolates were identified to the species level by a combination of conventional and molecular techniques, comprising examination of macro- and micro-morphology (Fig. 1) and the internal-transcribed spacer (ITS) rDNA region sequencing with ITS1 (5′-TCCGTAGGTGAACCTGCGG-3′) and ITS4 (5′-TCCTCCGCTTATTGATATGC-3′) primer pairs [24]. All nucleotide sequences were deposited in the GenBank database (Table 1). Samples were maintained at − 80 °C in Sabouraud dextrose broth (SDB; Oxoid, Hampshire, UK) supplemented with 15% glycerol and stored in the Department of Veterinary Microbiology, University of Life Sciences in Lublin, Poland.

In vitro tests of susceptibility to the antifungal compounds were performed for all the tested isolates according to Clinical and Laboratory Standards Institute (CLSI) document M38, 3^rd^ edition [25]. All chemicals used in this method were purchased from Sigma-Aldrich (Saint Louis, Missouri, USA), if not stated otherwise, and were of analytical grade. Stock solutions of the tested drugs were prepared in DMSO. Assay microtiter plates with 96 U wells were incubated for 72 h at 30 °C and then read spectrophotometrically using a Varioskan LUX multimode microplate reader (ThermoFisher) at the 530 nm wavelength (*λ*). The endpoint for the minimal inhibitory concentration (MIC_80%_) was the antifungal concentration at which prominent inhibition of growth, i.e., ≤ 80% of that of the control was observed. All isolates were tested in triplicate on different days. Prior to *ERG* gene expression analysis the inoculum, i.e., clinical isolates of *T. verrucosum* were grown for 14 days in Sabouraud glucose broth (BioMaxima, Lublin, Poland) at 25 °C. Next, they were homogenized and diluted to a final density of 1 × 10^3^ to 3 × 10^3^ CFU/ml. The suspension was exposed to fluconazole for 15 h (Sigma-Aldrich, Missouri, USA). The fluconazole was diluted in RPMI1640 medium (Sigma Aldrich, Missouri, USA) at the same concentration as the MIC determined for each sample. Drug-free controls were maintained for 15 h in the same medium without previous exposure to fluconazole.

### RNA extraction and synthesis of cDNA

RNA extraction was performed using TRIzol reagent. Prior to the RNA extraction, 0.5 ml of TRIzol containing the fungal cell suspension was vortexed for 6 min. Afterwards, 0.2 ml of chloroform (Sigma-Aldrich, Missouri, USA) was added, mixed for 15 s, and centrifuged at 12,000 × g for 15 min at 4 °C. Next, the transparent phase was transferred to a clean tube containing 0.5 ml of isopropyl alcohol, and RNA was precipitated out of the mixture at − 20 °C overnight. The next day, the samples were centrifuged at 12,000 × g for 30 min at 4 °C. The precipitated RNA was washed twice with 1 ml of cold 70% ethyl alcohol and centrifuged at 12,000 g for 5 min at 4 °C. The samples were dried at room temperature in vacuum conditions and the RNA was dissolved in ultrapure RNase-free water. The total RNA was treated with DNase I (Sigma-Aldrich, Missouri, USA). The quantity and purity of the RNA were assessed both spectrophotometrically based on the A260/A280 and A260/A230 ratios measured by NanoDrop 2000 (Thermo Fisher scientific, USA) and visually during electrophoresis performed in 1% denaturing agarose gel. Two micrograms of the RNA obtained were reverse-transcribed into cDNA to a final volume of 40 μl using RevertAid™ Reverse Transcriptase (Thermo Fisher Scientific).

### ERG gene expression

Quantitative reverse transcription PCR (qRT-PCR) was carried out with a StepOnePlus (Applied Biosystems, Foster City, USA) system using the SYBR Green GoTaq qPCR Master Mix (Promega, Madison, USA) to determine the relative expression levels of mRNA transcripts of *ERG3, ERG6*, and *ERG11* genes in the fungal samples using the housekeeping gene *GAPDH* as a reference. All PCRs were performed as follows: denaturation at 95 °C for 10 min, followed by 40 cycles of 15 s at 95 °C, 30 s at 58 °C, and 30 s at 72 °C. The sequences of primers of the *ERG3*, *ERG6*, *ERG11*, and *GAPDH* genes used in the qRT-PCRs are given in Table 2. The primers were designed in the Primer 3 (http://simgene.com/Primer3) Web-based software using sequences available in NCBI (National Center for Biotechnology Information) database. The analyses of the gene expression were performed using the 2^(−ΔΔCT)^ method of relative quantification [26].

**Table. 2:** Gene-specific primers used for real-time RT-PCR assays

Gene	Product	Primers (5′-3′)	Amplicon length (bp)
*ERG3*	Sterol C-5 desaturase	F: GACGGATACGTTCAGTCGCT R: GCCGTCGTGGATGAAGATTG	120
*ERG6*	Sterol C-24methyltransferase	F: CGAACACCTTTGATGCGGTG R: TTCATACACGCCAAACACGC	119
*ERG11*	Lanosterol 14-α demethylase	F: CTGACCCAGCCCATCAACAT R: TGGGGATGTTGCTCTTCACG	112
*GAPDH*	Glyceraldehyde-3-phosphate dehydrogenase	F: AACGGCTTCGGTCGTATTG R: TATTCGGCGTATTTGGTCTCA	110

*F* forward, *R* reverse

### Statistical analysis

The results are presented as the mean ± SEM. All values were compared using either Student’s *t* test or one-way ANOVA followed by Bonferroni’s post hoc test. Student’s *t* test was used to analyze *ERG* gene expression between fluconazole-sensitive and -resistant isolates. While ANOVA was applied to compare the relative expression of *ERG3*, *ERG6* and *ERG11* in resistant strains exposed to fluconazole. Statistical significance was considered when the *p* value was < 0.05. Statistical analyses were made using the R software (R Core Team, Auckland, New Zealand).

## Results

The fluconazole-resistant *T. verrucosum* isolates used in this study showed MIC values ranging from 8 to 128 μg/ml (Table 1). The geometric mean MICs for the human and animal isolates were 44.8 μg/ml and 58.5 μg/ml, respectively. Tinea capitis (60%) and typical kerion celsi (30%) symptoms caused by fluconazole-resistant *T. verrucosum* were the most frequently reported disease in humans (Table 1). In turn, isolates with the highest MIC values for fluconazole (128 μg/ml) were detected in cattle and in one case of human kerion celsi. Cattle isolates with MIC values of fluconazole in the range of 0.25–0.5 μg/ml were used as control fluconazole-susceptible strains of *T. verrucosum*.

**Fig. 2:**
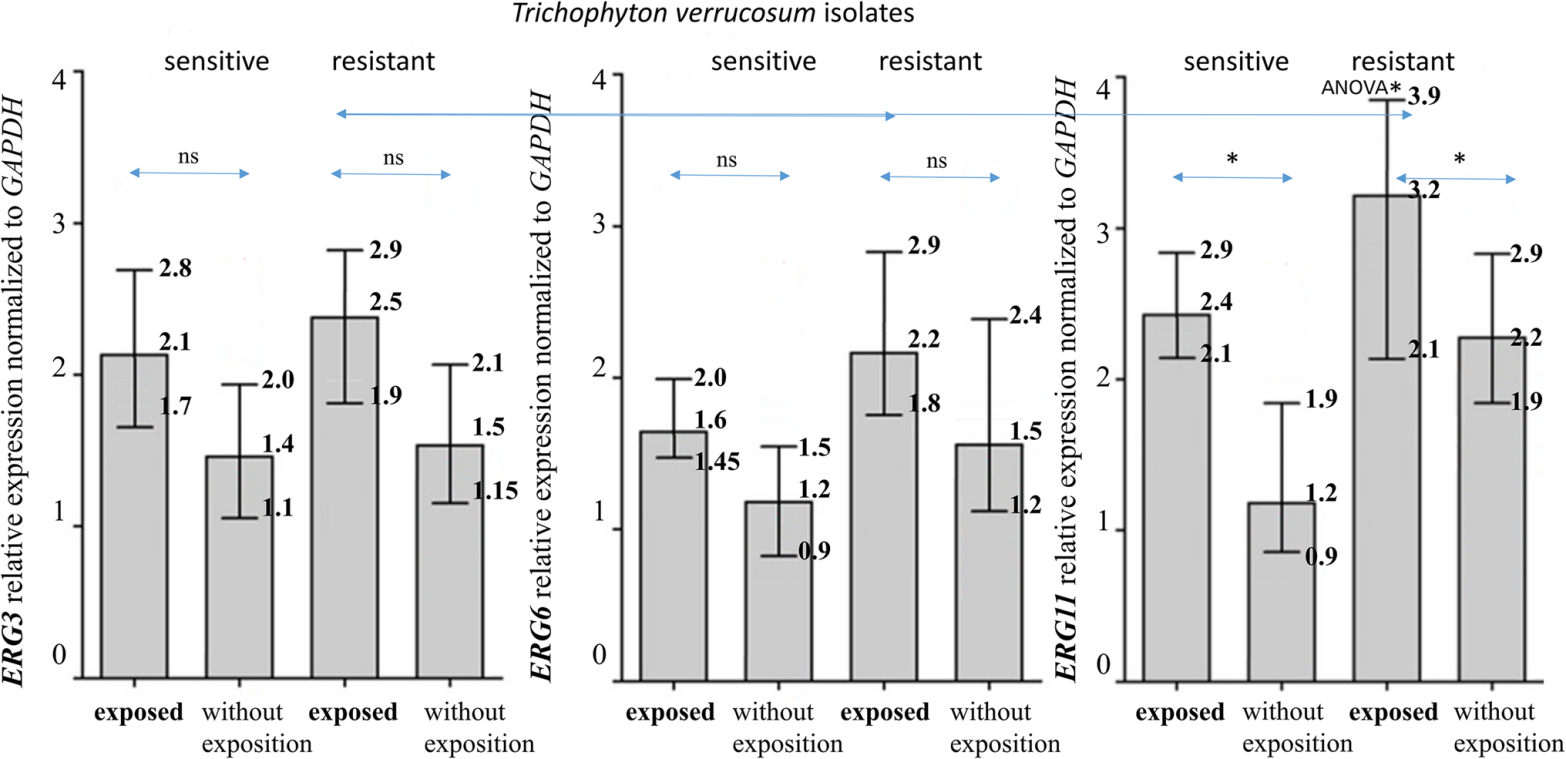
*ERG* gene expression based on the antifungal susceptibility profile. Fungal samples of fluconazole-resistant and -sensitive *Trichophyton verrucosum* were grown for 14 days in Sabouraud glucose broth and exposed- or non-exposed to the drug for 15 h. The RNA was extracted and real-time PCR analysis was performed to evaluate mRNA expression of the *ERG3* (**a**), *ERG6* (**b**), and *ERG11* (**c**) genes. *GAPDH* was used as a housekeeping gene. Ns no statistical significance and * statistical significance tested by Student’s *t* test between bars. ANOVA* statistical significance tested by ANOVA followed by Bonferroni’s post hoc test between relative expression of *ERG3*, *ERG6*, and *ERG11* in resistant strains exposed to fluconazole

Additionally, based on the quantitative reverse transcription PCR (qRT-PCR), different levels of expression of the *ERG3*, *ERG6*, and *ERG11* genes were shown in the pool of the clinical isolates of *T. verrucosum*. The exposure to fluconazole induced a higher level of *ERG3, ERG6*, and *ERG11* gene expression in the cells of the resistant isolates compared to the sensitive ones (Fig. 2). However, despite these results, there was no statistical difference in the levels of *ERG3* and *ERG6* gene expression between the susceptible and resistant isolates after and without the exposure to fluconazole. The increase in the level of expression of these genes after the exposure to fluconazole was only 0.2 to 0.4 times higher in the phenotypically resistant isolates than in the susceptible ones. Interestingly, a statistically significant increase in the level of *ERG11* gene expression was observed after and without the exposure to fluconazole in both the resistant and susceptible isolates. Moreover, the level of expression of the *ERG11* gene in the fluconazole-resistant clinical isolates was statistically higher than for the *ERG3* or *ERG6* genes in these isolates.

## Discussion

The knowledge of the mechanisms that allow dermatophyte cells to develop drug resistance is essential for designing novel drugs and alternative therapeutic approaches [27]. This is even more important considering the infections by zoophilic dermatophytes in humans that are commonly noted in European countries [8, 28, 29], including recalcitrant dermatophytosis [27]. The frequency of infections caused by *T. verrucosum* varies from 3.1% in Spain, through 1.8% in Greece, and 1% in Poland to 0.01% in the Czech Republic [9, 30].

Ergosterol is a fundamental component of fungal cell membranes; therefore, many drugs, like polyenes, were designed to directly bind with this final product or, e.g., azoles, to disturb and inhibit the function of enzymes involved in its synthesis [14]. It is commonly known that the efficacy of azoles is the result of both the depletion of ergosterol in the plasma membrane and the subsequent accumulation of toxic sterol 14-α-methylated intermediates, ultimately leading to cell growth arrest [16, 19]. In consequence of perturbation of ergosterol biosynthesis during azole-treatment, the expression of some genes belonging to various sterol pathways are up or downregulated as a compensation mechanism [31, 32]. Lanosterol 14-α demethylase encoded by the *ERG11* gene is the crucial enzyme [14]. An important role in this compensatory phenomenon in dermatophyte cells is also attributed to sterol C24-methyltransferase transferase and sterol C5-desaturase, i.e., products of *ERG6* and *ERG3* gene expression, respectively [31]. Here, we used the qRT-PCR method to determine the expression levels of three specified *ERG* genes to shed more light on the susceptibility and resistance to fluconazole of *T. verrucosum* strains. Since the mechanisms of azole resistance in zoophilic dermatophytes are not fully understood, compared to anthropophilic species, studies showing the resistance mechanism in *T. verrucosum* strains related to the expression of genes involved in ergosterol biosynthesis are particularly important and necessary.

Our study has demonstrated that exposure to fluconazole of *T. verrucosum* cells increased the expression levels of the *ERG3, ERG6*, and *ERG11* genes both in the susceptible and resistant isolates. However, only the mRNA fold change in the *ERG11* gene achieved a statistically significantly higher level in isolates with phenotypic resistance to fluconazole than in the case of the susceptible control strains. In literature, there are only fragmentary results regarding dermatophytes in this context. Diao et al. [31] found that the *ERG11* gene was upregulated 13.55-fold in *T. rubrum* after exposure to itraconazole. Similar data obtained in investigations of isolates of *Candida glabrata* demonstrated that treatment with an azole drug, i.e., voriconazole, also increased the level of *ERG3*, *ERG6*, and *ERG11* gene expression [33]. The ability of fluconazole to upregulate *ERG11* gene expression in C*. tropicalis*, *C. glabrata*, and *C. krusei* cells was examined by many authors [33–35]. However, Alizadeh et al. [36] demonstrated differential expression of the *ERG11* gene in resistant *Candida albicans* only after exposure to fluconazole. Nonetheless, *ERG* gene upregulation may play a role in azole resistance in fungi, regardless of the phylogenetic group.

It is worth noting that *ERG11* is the most statistically significantly overexpressed *ERG* gene in the pool of fluconazole-resistant *T. verrucosum* isolates. Our studies show that the *ERG3* and *ERG6* genes seem to be less important than *ERG11* for the phenomenon of azole resistance in zoophilic dermatophytes. These conclusions also raise some doubts in relation to research conducted on yeast-like fungi. Geber et al. [37] revealed that the regulatory mechanisms involved in the expression of these genes are interrelated, and deletion of *ERG3* in *Candida glabrata* was associated with simultaneous upregulation of the *ERG11* gene. Similarly, deletion of *ERG11* was associated with *ERG3* gene upregulation. Furthermore, in studies on *Saccharomyces cerevisiae*, Bammert and Fostel [38] documented global *ERG* gene upregulation in response to azole, terbinafine, and amorolfine treatment and mutations in the *ERG1*, *ERG11*, *ERG6*, *ERG2*, and *ERG5* genes. However, Henry et al. [35] demonstrated that the increased *ERG11* expression levels in drug-treated *C. albicans* quickly decreased upon removal of the antimycotic. In turn, Lopez-Ribot et al. [39] and Fernandes et al. [40] showed that, in the case of some species of azole-resistant *Candida* spp. treated with fluconazole, *ERG* genes are overexpressed but not genes encoding specific efflux pumps. In the study presented here, the participation of commonly known efflux pumps in the generation of the observed resistance phenotypes among *T. verrucosum* strains cannot be excluded. This seems very likely and will be the subject of further research, considering the fact that, unlike other azoles, fluconazole can be a good substrate for specific efflux pumps, as described elsewhere [41]. All the data presented here indicate a possible role of *ERG* genes in resistance to azole drugs, also in dermatophytes. However, this research should be continued.

Finally, our results have shown one of the possible mechanisms of resistance to fluconazole among *T. verrucosum* isolates, which involves the maintenance of high levels of expression of *ERG* genes after drug exposure. Further studies are necessary to determine whether other mechanisms also contribute to the maintenance and transmission of azole-resistant isolates. The gene expression profiling seems to be a useful tool for understanding the mechanisms of action and acquiring resistance to antifungal agents by zoophilic dermatophytes.

## Data Availability

All data generated or analyzed during this study are included in this published article.
